# Lobster Supply Chains Are Not at Risk from Paralytic Shellfish Toxin Accumulation during Wet Storage

**DOI:** 10.3390/toxins13020129

**Published:** 2021-02-09

**Authors:** Alison Turnbull, Andreas Seger, Jessica Jolley, Gustaaf Hallegraeff, Graeme Knowles, Quinn Fitzgibbon

**Affiliations:** 1 South Australian Research and Development Institute, GPO Box 397, Adelaide, SA 5001, Australia; andreas.seger@utas.edu.au (A.S.); Jessica.jolley@sa.gov.au (J.J.); 2 Institute for Marine and Antarctic Studies, University of Tasmania, Private Bag 129, Hobart, TAS 7001, Australia; gustaaf.hallegraeff@utas.edu.au (G.H.); quinn.fitzgibbon@utas.edu.au (Q.F.); 3 Animal Health Laboratory DPIPWE Tasmania, 165 Westbury Rd, Prospect, TAS 7250, Australia; graeme.knowles@dpipwe.tas.gov.au

**Keywords:** lobster health, toxic algae, *Alexandrium*, *Jasus edwardsii*

## Abstract

Lobster species can accumulate paralytic shellfish toxins (PST) in their hepatopancreas following the consumption of toxic prey. The Southern Rock Lobster (SRL), *Jasus edwardsii*, industry in Tasmania, Australia, and New Zealand, collectively valued at AUD 365 M, actively manages PST risk based on toxin monitoring of lobsters in coastal waters. The SRL supply chain predominantly provides live lobsters, which includes wet holding in fishing vessels, sea-cages, or processing facilities for periods of up to several months. Survival, quality, and safety of this largely exported high-value product is a major consideration for the industry. In a controlled experiment, SRL were exposed to highly toxic cultures of *Alexandrium catenella* at field relevant concentrations (2 × 10^5^ cells L^−1^) in an experimental aquaculture facility over a period of 21 days. While significant PST accumulation in the lobster hepatopancreas has been reported in parallel experiments feeding lobsters with toxic mussels, no PST toxin accumulated in this experiment from exposure to toxic algal cells, and no negative impact on lobster health was observed as assessed via a wide range of behavioural, immunological, and physiological measures. We conclude that there is no risk of PST accumulation, nor risk to survival or quality at the point of consumption through exposure to toxic algal cells.

## 1. Introduction

The Southern Rock Lobster (*Jasus edwardsii* Hutton) is sold in high value live export fisheries in Tasmania, Australia, and New Zealand worth AUD 97 M and AUD 268 M, respectively [1,2]. Lobsters are known to accumulate paralytic shellfish toxins (PST) during blooms of PST-producing algal species in Tasmanian and New Zealand coastal waters [3,4]. The causative alga in Tasmania is *Alexandrium catenella* (Whedon and Kofoid) Balech, whilst New Zealand blooms may be *A. minutum* Halim, *A. pacificum* Litaker (previously identified as *A. catenella*) and *Gymnodinium catenatum* Graham [5,6]. The toxins accumulate in the lobster hepatopancreas via the consumption of contaminated prey but are not found in the tail meat [7,8].

Whilst there is no Australian or New Zealand food standard for PST in lobster, several key export markets such as China and Hong Kong stipulate a maximum level of 0.8 mg saxitoxin (STX) equivalents kg^−1^. Furthermore, human health risk assessment has shown a risk of illness for consumers if consuming large quantities of lobster hepatopancreas. This risk is significantly reduced if the bivalve regulatory level of 0.8 mg STX equivalents kg^−1^ is applied [9]. In both Tasmania and New Zealand, the public health and market access risks associated with PST in *J. edwardsii* are managed during high-risk periods through weekly or fortnightly biotoxin monitoring of bivalve sentinel species in coastal waters, followed by direct monitoring of lobster hepatopancreases when bivalves indicate risk.

**Figure 1:**
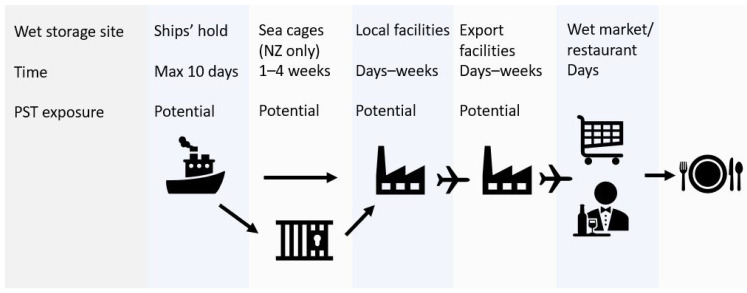
Supply chain for *J. edwardsii* from Tasmania and New Zealand to Asian markets, showing wet storage and potential paralytic shellfish toxin (PST) exposure sites. Biotoxin risk monitoring occurs in coastal waters prior to entry into the supply chain.

The *J. edwardsii* supply chain is focused on live seafood markets in Asia. Wet storage is employed to maintain animal quality and facilitate maximum price return during market fluctuations. Animals are moved into wet storage immediately after capture and remain in specialised holding facilities as they move through the supply chain [10], as depicted in Figure 1. The seawater used for wet storage is sourced from local coastal waters. 

Wet storage times may range from a few days to several weeks depending on the time of year and market demands. If PST-producing algal blooms are present in coastal waters, these algae will be inadvertently pumped into holding compartments on-vessel, in sea cages (New Zealand only), or in local/export holding facilities. As a result, *J. edwardsii* may be exposed to PST in the supply chain, post regulatory monitoring programs. 

Crustacean gills have multiple functions, such as ionic transport mediating haemolymph osmoregulation, acid–base balance, and ammonia excretion. Heavy metal accumulation in crustaceans also occurs via the gills [11]. No studies have examined the potential for PST uptake in lobsters when directly exposed to toxic algae. Furthermore, lobsters may be in the supply chain for significant periods of time and subjected to more than one period of emersion during transport. To maximise value in the market, lobsters need to survive the rigours of international transport and thus it is integral that they start their journey in strong health [12]. Whilst a recent study showed no impact of PST feeding-related accumulation on *J. edwardsii* health [13], no studies have so far examined the sensitivity of lobster gill cells to the superoxide radicals, exudate phycotoxins, and fatty acids that are known to be produced by toxic *Alexandrium* spp. and to have a deleterious impact on fish gill cells [14,15].

A range of indicators have been used to assess stress and predict mortality in commercially-important crustacean species [12]. In this study, we have taken a holistic approach to determine the impact of PST, examining both whole organism indicators (survival, nutritional condition, reflex, behaviour, and health) and cellular indicators (haemolymph immunity, biochemical parameters, and gill histopathology).

Commercial operators assess *J. edwardsii* health during grading using a subjective vitality scale which is based on lobster reflex and behavioural responses [16]. Reflex actions are consistent, involuntary, nearly instantaneous responses to stimuli which can reliably indicate crustacean whole-body health status independently of animal size, strength, motivation and gender [12]. Crustacean reflex scores have been previously used to provide an accurate indicator of crustacean performance in supply chain studies [12,17,18,19,20]. Other commonly used methods to assess lobster gross or whole-body performance include survival and nutritional condition [16,19,21,22]

Physiological indicators for the assessment of the health and vitality of crustaceans commonly include immune responses (raised bacteraemia levels and changes in haemocyte counts [21,23,24,25,26]; and haemolymph concentration of ions (e.g., potassium, sodium, magnesium, calcium, bicarbonate, pH), metabolites (e.g., ammonia, urea, glucose, lactate) and hormones (e.g., crustacean hyperglycaemic hormone) [21,25,27,28].

The present study aimed to determine if *J. edwardsii* could accumulate PST through exposure to PST-producing algae, and to ascertain whether direct exposure to these algae could impact lobster health and vitality. In a biosecure experimental aquaculture facility, lobsters were exposed to field-relevant concentrations of toxin-producing *A. catenella* algae for 21 days. PST concentrations in the hepatopancreas were measured, as well as a range of measures commonly employed to assess survivability and lobster health.

## 2. Results

### 2.1. Stocking Animals

Lobsters arrived in good condition, with vitalities on receipt ranging from 4–5 (maximum score possible is 5). There was no significant difference in the lobster harvest wet weights (514 ± 34 g; *p* value = 0.59) or carapace lengths (104 ± 3 mm; *p* value = 0.37) between each treatment group. No lobsters moulted during the experiment, but two lobsters in the high exposure treatment groups died; one on day 10, and one on day 11.

### 2.2. Specific Feed Intake

**Figure 2:**
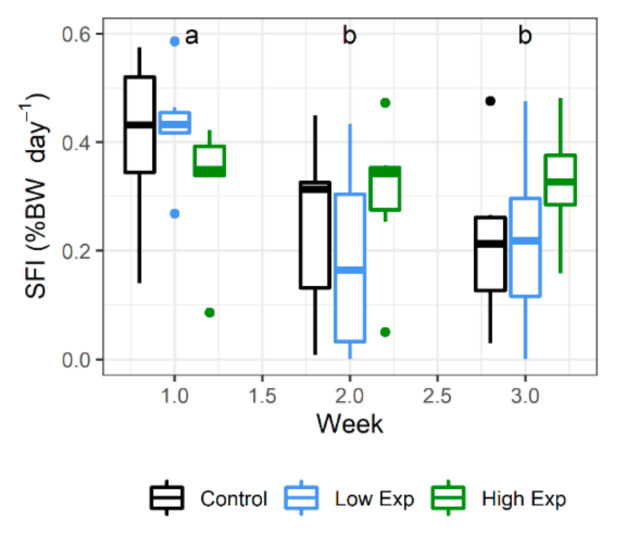
Weekly specific feed intake (SFI) of *J. edwardsii* lobsters exposed to 0, 1 × 10^5^ or 2 × 10^5^ cells of *A. catenella* per litre of tank water (control, low, or high exposure (exp) groups, respectively) across three weeks of exposure. Weeks where SFI is not significantly different share the same letter. BW: body weight.

Lobsters from all treatment groups ate well during the experiment, with no significant difference in the specific feed intake (SFI) between treatment groups. Feed consumption decreased during the experiment, with SFI during weeks 2 and 3 being significantly less than that at week 1 (Figure 2; *p* value = 0.0009). 

### 2.3. PST Accumulation

**Figure 3:**
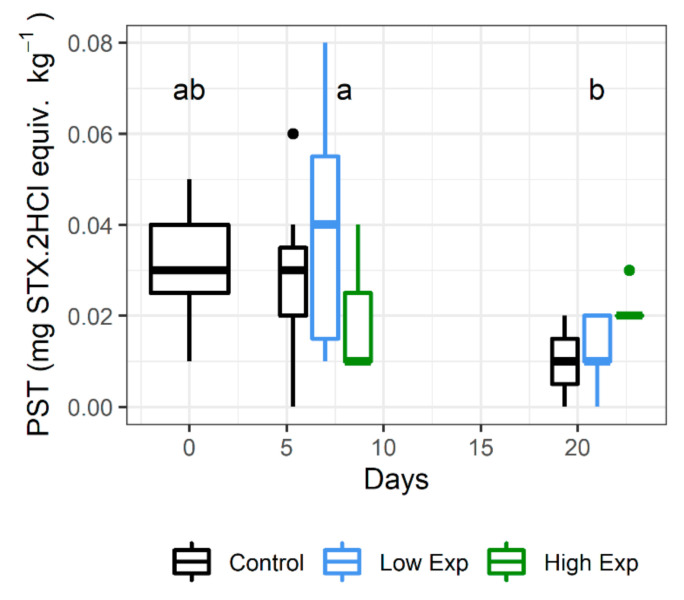
PST concentrations in the hepatopancreas of *J. edwardsii* lobsters harvested on days 0, 7 and 21 after exposure to 0, 1 × 10^5^ or 2 × 10^5^ cells of *A. catenella* per litre of tank water (control, low, or high exposure groups respectively). Days where the PST concentration is not significantly different share the same letter. STX: saxitoxin.

Lobsters had low concentrations of PST in the hepatopancreas on receipt (mean 0.03 ± 0.01 mg STX.2HCl equiv. kg^−1^). Lobsters harvested on day 21 had significantly lower PST than those harvested on day 7 (*p* value = 0.001), but not on day 0 (*p* value = 0.53; Figure 3; Supplementary Table S1). There was no significant difference between PST levels among treatment groups (*p* value = 0.64).

### 2.4. Lobster Health Responses

There was minimal difference between treatment groups across a wide range of behavioural, immunological, and physiological parameters measured, as summarised in Table 1. The means and standard deviations of continuous variables are provided in Supplementary Table S2.

### 2.5. Behavioural Responses

No significant difference between treatments or across days in the experimental system was seen in any of the behaviour measures tested (other than SFI, as discussed above). Lobster vitality remained high throughout the experiment, with 36 animals scoring the maximum vitality score (5), seven scoring a vitality of 4, and three scoring a vitality of 3. All lobsters responded quickly to being placed ventrum-up, righting within 28 s. Impairment of reflexes was low, with 32 lobsters showing impairment of three or less reflexes (Supplementary Table S2). Reflex impairment scores were significantly related to vitality scores (*p* value = 0.0003; Supplementary Table S3).

### 2.6. Immune Health Response

**Figure 4:**
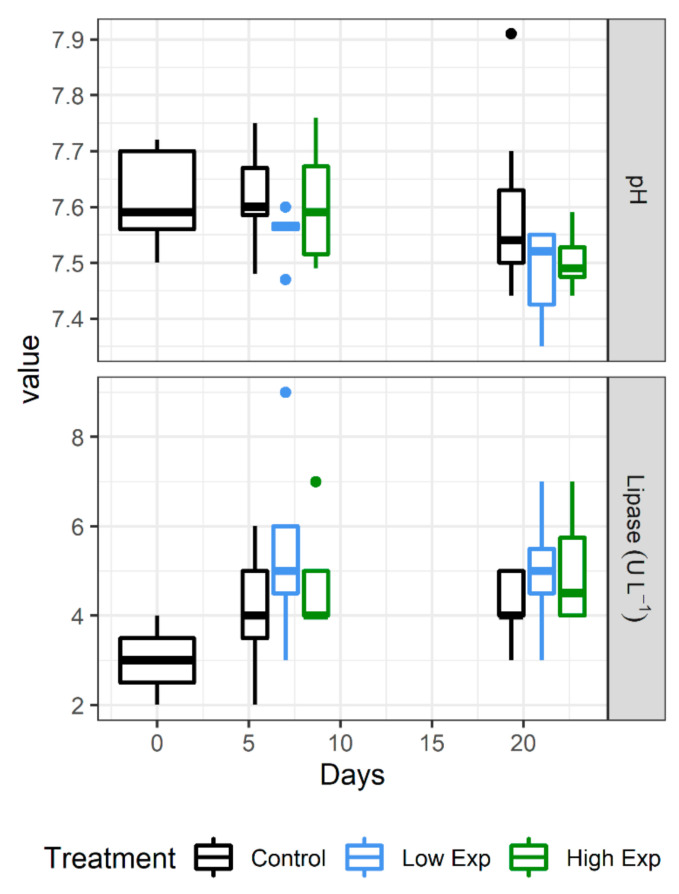
Haemolymph biochemical parameters that showed significant differences between *J. edwardsii* treatment groups. Low exposed (dark grey) lobsters had significantly lower pH than the control group (*p* value = 0.02). Both high and low exposed lobster groups had significantly higher lipase than the control group (*p* values = 0.03 and 0.02, respectively).

There was no change to bacteraemia concentrations or the prevalence of necrosis between treatment groups or days, however haemocyte counts did increase significantly across all treatment groups at day 21 compared to days 0 and 7 (Table 1, Figure 4; *p* values = 0.0007 and 0.002, respectively).

### 2.7. Nutritional Response

There were no significant differences between the nutritional indicators of Brix and hepatopancreas index across treatments or across days (Table 1, Supplementary Table S3).

### 2.8. Haemolymph Biochemical Response

Of the range of electrolytes, minerals, metabolites, and enzymes examined, only pH and lipase showed any significant difference between treatments (Figure 4). The low exposure treatment group showed significantly lower pH than the control group (*p* value = 0.02), whilst both low and high exposure groups showed significantly higher lipase concentrations than the control group (*p* value = 0.03 and 0.02, respectively).

**Figure 5:**
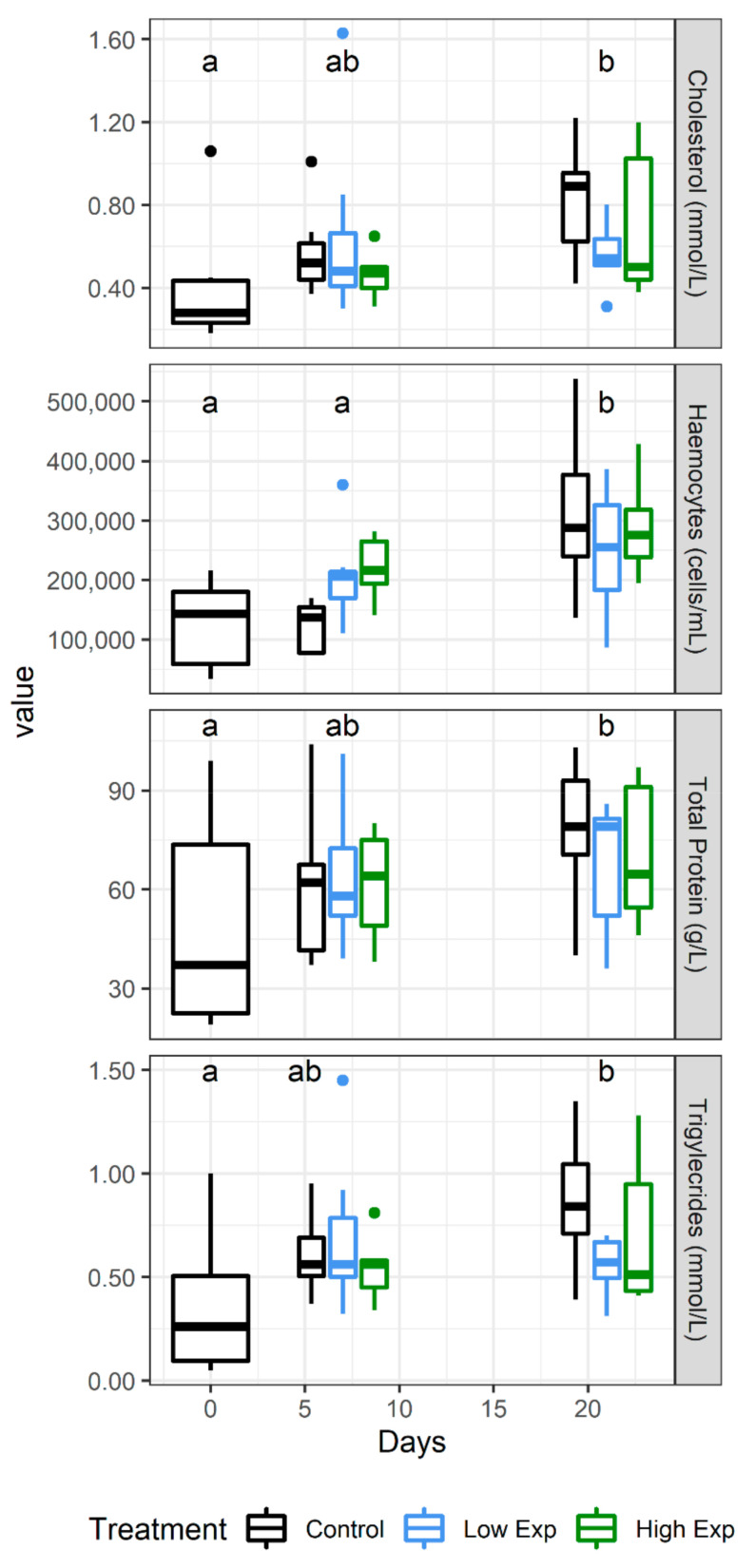
*J. edwardsii* haemolymph parameters that changed significantly over the course of the experiment in control (black), low (blue), and high (red) algal exposure treatments (0, 1 × 10^5^, 2 × 10^5^ *A. catenella* cells/L, respectively). Days which are not significantly different from each other share the same letter.

No difference was observed in electrolyte, mineral, or enzyme levels across the course of the experiment, but the metabolites cholesterol, total protein, and triglycerides all increased significantly across the duration of the experiment (Figure 5).

### 2.9. Histopathological Findings in Gills

**Figure 6:**
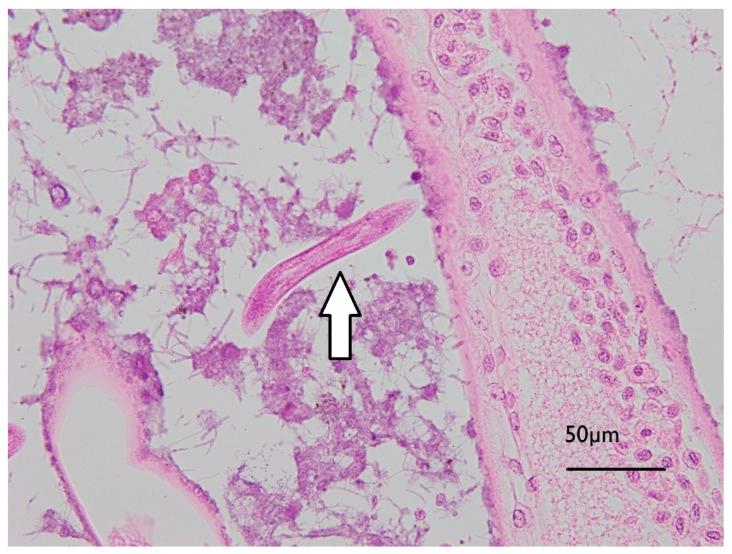
Micrograph of *J. edwardsii* gill filament with biofouling (mixed bacterial colonies, including Leucothrix-like organisms, adhered to filaments) and a free-living nematode between filaments as indicated by the arrow.

Initial examination of gill filaments from the six high exposed and three of the control animals showed no differences in histopathological findings, so no further examinations of other lobster gills occurred. Diffuse pooling of haemolymph was observed in the lamellae and central axis of all gills examined, consistent with agonal change. All gills examined also showed multifocal deposits of rod and/or filamentous bacteria and low to moderate numbers of ciliated protozoa. Low numbers of free-living larval nematodes were found between lamellae on three of the six samples of gill tissues examined from exposed lobsters, and one of the three gill tissues from control lobsters. The gills of two of the high exposure lobsters and one control lobster showed microvesicles in reserve inclusion cells (Figure 6), likely storing lipids [29].

## 3. Discussion

No uptake of PST was detected in *J. edwardsii* exposed to high but field-relevant concentrations of *A. catenella* in an experimental setting over a three-week period. This was in striking contrast to significant PST accumulation in the lobster hepatopancreas (reaching a maximum of 9.0 mg STX.2HCl equiv. kg^−1^) observed in parallel experiments involving feeding lobsters with toxic mussels [8], and uptake of PST by abalone when exposed to toxic algal cells in a similar experiment to the current study [30]. In the latter experiment, abalone were exposed to the same highly toxic strain of *A. catenella* (AT. TR/F) at the same level of the high exposure group in this experiment (2 × 10^5^ cells L^−1^). Abalone were able to accumulate up to 128 µg STX.2HCl equiv. kg^−1^ in this experiment, although it is unknown if this accumulation occurred across the gills, epipodium, via the viscera, or a combination of these routes.

Furthermore, the minimal impact on lobster health, demonstrated in this experiment across a range of organismal and cellular levels, indicates that there is no detrimental effect on the survivability and vitality of these animals as a result of exposure to toxic cells. Minimal whole organism and cellular responses were observed in *J. edwardsii* following the accumulation of high levels of PST in their hepatopancreas [13] and when exposed to toxic cells (current experiment), indicating that lobsters are relatively resistant to the action of PST. However, this response is in contrast to significant histopathology and mortality experienced by Blue mussel and Pacific oyster larvae when exposed to extracellular exudates of the same Tasmanian *A. catenella* strain at equivalent cell concentrations of 100 to 1000 cells mL^−1^ [31].

The present study exposed lobsters to aliquots of cultured algae and replicated environmental conditions where animals would be exposed to both cells and cell exudates. The toxic cells were presented to the algae at the highest level recorded from the Tasmanian blooms [3]. It is likely that toxins in wet storage would be equal to or less than those found in the field, as wet storage areas either draw directly from coastal waters in a continuous flow through systems or recirculate sea water through filtration and sedimentation systems to maintain water conditions.

The cultured *A. catenella* strain (AT. TR/F) was originally isolated from a bloom on the east coast of Tasmania and contained up to 21.2 pg STX.2HCl equiv. cell^−1^, a relatively high PST cell quota from cultured algae [32,33,34,35]. The toxin profiles of the *A. catenella* cells were predominantly di-sulfated carbomoyl (C) 1,2 and gonyautoxin (GTX) 1,4, with minor levels of C3,4, neosaxitoxin (Neo), GTX2,3, STX, decarbamyolated gonyautoxin (dcGTX) 2,3, and GTX5,6 (see Section 4.2 below). These analogues are the same as those found in toxic shellfish from the east coast of Tasmania [36,37] and are thus considered representative of the Tasmanian *A. catenella* blooms. The same PST analogues are also found in New Zealand *A. pacificum*, *A. minutum* and *G. catenatum* isolates, although toxin proportions vary [5,38]. In particular, the proportion of C3,4 toxins tends to be higher in *A. pacificum* isolates, Neo and STX higher in *A. minutum* isolates, and C3,4 and GTX2,3 higher in *G. catenatum* isolates. Absolute concentrations present at any time will vary with the cell number and toxin content of the cells, and the amount of cell exudate.

Bioactive exudates from ichthyotoxic species have been demonstrated to have harmful impacts on the gills of adult Pacific oysters [39] and a range of fish species [15,40,41]. Compromised gills show necrotising degeneration of the epithelium of the secondary lamellae and sloughing and swelling of the primary lamellar epithelium with congestion of branchial vessels [42,43,44]. None of these effects were seen in the lobster gills exposed to high concentrations of *A. catenella* in this experiment.

A wide range of indicators used to predict the health of lobsters throughout the supply chain were assessed in this study. The only characteristics that demonstrated significant differences between treatment groups was haemolymph pH and lipase concentration. A difference in pH was found between control and low exposure groups, with the control group showing higher pH. A decrease in pH is a common stress response in lobsters caused by respiratory and metabolic acidosis. Other studies found a decrease in pH associated with emersion and high temperature [25,45,46,47]. Given that the high exposure group was not significantly different to the control and low exposure group, it is unlikely this difference was caused by the exposure to *A. catenella*.

Lipase plays an important role in the digestion of fats. The increase seen between the control and exposed groups of lobsters was influenced by the relatively low level of lipase measured in the control group at the start of the experiment. No significant differences were seen between control and the two treatment groups if the lobsters on day 0 were excluded from the analysis (*p* value = 0.21). This observed difference could be related to the improved nutritional condition of the lobsters across the experiment, as shown by significant increases in cholesterol, protein, and triglyceride levels. This increase in nutritional status was also detected in similar experiments involving feeding *J. edwardsii* mussels to excess daily [13] and was also associated with a similar decrease in feed intake during the course of the experiment as seen here.

The only immune response indicator that showed variation across the experiment was haemocyte levels, which increased with time in the system in all treatment groups. Other studies looking at stress in lobster supply chains have found varying results; some have found an increase in haemocyte levels with starvation, capture, emersion, storage and transport [21,25,26]; and others have found a decrease [18,24,48,49,50]. No difference in haemocyte levels was seen in a similar experimental study looking at the impact of PST accumulation on *J. edwardsii* health [13]. It is possible that the increase over time in all treatment groups may be related to the static experimental system.

In conclusion, we have conducted the first reported experiment to examine the uptake of PST by lobsters during exposure to PST-producing algal cells, as would potentially occur in lobster supply chains. The algal culture used was highly toxic, produced a range of commonly found PST analogues, and was presented at relatively high concentrations. The lobsters were exposed to toxic cells for three weeks, longer than would normally be experienced in the *J. edwardsii* supply chain in Tasmania and New Zealand. From the lack of uptake of PST in lobsters during this study and the lack of impact on animal health, we conclude that the wet storage of lobsters in coastal waters contaminated with the PST-producing algae typically found in Tasmania and New Zealand, as occurs in the *J. edwardsii* supply chain, does not pose a human health risk, nor an animal health risk. Therefore, no market access or risk to commercial returns through ill health exists from this practice.

## 4. Materials and Methods

### 4.1. Experimental System

The experimental system used is described in detail in Turnbull et al. [8], with the exception that in this case, a static system was employed with total daily water exchange. Briefly, 450–600 g adult *J. edwarsdii,* (n = 48 male and 1 female) were sourced directly from South Australian fishing vessels from a mix of shallow and deep habitats with no known bloom activity. The lobsters were transported to the South Australian Aquatic Biosecurity Centre at Roseworthy, where they were held in individual 30 L tanks maintained between 13.1 and 16.3 °C, salinity of less than 37 ppt, and pH between 7.7–8.2 (supplementing the seawater supply with bicarbonate soda as necessary). Water quality was maintained using pre-conditioned sponge biofilters. Dissolved oxygen was >90% saturation and 10 lumens of light was provided on a 12:12 h light:dark cycle. Lobsters were acclimated for 7 days prior to exposure.

### 4.2. Algal Cultures

Batch cultures of *A. catenella* strain AT.TR/F (previously known as *A. tamarense* group 1; isolated from Triabunna, Tasmania at the Institute for Marine and Antarctic Studies, Hobart, Australia) were cultivated in 15 L carboys following the method of Seger et al. [30]. Cultures were maintained in sterile filtered seawater (0.22 µm) supplemented with modified GSe nutrient concentrations (final media = 3/4 GSe nutrients, 5 mM sodium bicarbonate and 7.5 pM H_2_SeO_3_ to replace the soil extract in the basal recipe). Cultures were grown at 18 ± 1 °C under 120 µmol photons m^−2^ s^−1^ of light supplied by low temperature light emitting diodes on a 12:12 h light:dark cycle. During the dark period, the carboys were gently aerated (0.15 L min^−1^) with ambient air, which was enriched with 1.5–2.5% (*v*/*v*) CO_2_ in the light.

Cultures used for the exposure experiments were in late exponential/early stationary phase (>2 × 10^7^ cells L^−1^, 2.5% CO_2_ aeration) and contained 3.5–21.2 pg STX.2HCl equiv. cells^−1^ [30]. Cell PST quotas were determined in parallel experiments by Seger et al. [27] to be 3.5–21.2 pg STX.2HCl equiv. cell^− 1^. Briefly, suspensions of toxic cells were concentrated from four different batches of the same monoclonal source culture in late exponential/early stationary phase through centrifugation. Extracts of these suspensions were produced by lysis and further centrifugation to remove cell fragments. The toxin content of the extracts was determined via LCMS-MS analysis by the Cawthron Institute, New Zealand, as described below. The average toxin profile on a molar basis was 55% C1, 2, 36% GTX1,4, 3% C3,4, 2% Neo, 2% GTX2,3, 2% dcGTX2,3, and small percentages (<2% each) of STX, GTX5, GTX6, and doSTX.

### 4.3. Lobster Treatments

Lobsters were fed to excess (3 in-shell blue mussels, *Mytilus galloprovincialis* Lamarck) at the same time each day during the 7 day acclimation period and for the course of the experiment. The mussels were sourced from Coffin Bay, South Australia and were confirmed to be free of toxins via LCMS-MS [51,52] at the Cawthron Institute, New Zealand. 

Lobsters were randomly allocated to three treatment groups: control (n = 21), low exposure (n = 14) and high exposure (n = 14). Each treatment group was further divided into harvest groups of seven replicates each. One control group was harvested on day 0, and the control, low, and high exposure groups were each harvested on days 7 and 21. Seven replicates were used in each group to minimise the number of experimental animals for ethical reasons whilst still allowing statistical rigour. The cell density of the *A. catenella* culture was determined daily via haemocytometer counts, and aliquots of culture were added to the low and high exposed lobsters at final concentrations of 1 × 10^5^ cells/L and 2 × 10^5^ cells/L, respectively, immediately after the morning water exchange each day.

### 4.4. Specific Feed Intake

The apparent feed intake (AFI) of lobsters were measured each week following the method of Fitzgibbon et al. [53]. Feed control tanks for the control, low, and high exposure groups were included in the random allocation, with no lobsters placed in these tanks. The feed control tanks each received 3 mussels at the same time as the lobster tanks each afternoon. Uneaten mussel meat from each tank (control and exposed animals, and feed control tanks) was collected at the beginning of each day. The shucked meat was frozen cumulatively over the period of a week. Subsequently, the uneaten mussel meat was dried at 105 °C for 24 h and weighed. The AFI of each lobster was calculated (dry weight of the uneaten food in the treatment tank subtracted from that of the respective control tank, divided by 7) and converted to SFI by dividing by the wet weight of the lobster.

### 4.5. Lobster Harvest Protocols

The harvest protocols and tests are described in detail in Turnbull et al. [13]. Briefly, lobster behavioural responses and tissue collection were conducted in the same order by the same researchers on each harvest day. Following behavioural measurements, lobsters were euthanised in an ice slurry, and then haemolymph samples (5–15 mL) were taken from the sinus under the right fifth leg joint. The animals were kept on ice overnight, then weighed, and the carapace length was measured prior to tissue dissection and collection. Gill tissue samples from each animal were immediately placed in Davidson’s fixative for 24 h, which was then replaced with 70% ethanol. Hepatopancreases were stored at −80 °C prior to PST analysis.

### 4.6. Behavioural Responses

Lobsters were first assessed for 7 reflex responses following Turnbull et al. [13]: primary and secondary pereopod lift, antennae and secondary antennal lift, and tail arch via photography whilst emersed; rapid (<1 sec) eye stalk return to normal after gently squeezing together; and rapid antennal touch of hand placed directly in front of immersed animal. Two behavioural responses were then assessed (righting response time measured by placing each animal ventrum-up in a tank of saltwater and recording the time taken to return to dorsum-up [54]; and vitality visually assessed on a lobster commercial operator 1–5 scale similar to that described by Spanoghe and Bourne [16]; 1 = dead; 2 = limp tail, no escape response, no response to handling; 3 = limp tail, some response to handling, i.e., leg movement; 4 = mostly alert, tail held erect; 5 = alert with vigorous escape behaviour). Each reflex response was scored (positive response = 0, negative response = 1) and summed into a reflex impairment score for each animal, as described by Stoner et al. [19]. Potential scores ranged from 0–7, with 0 indicating maximum vigour.

### 4.7. Immune Health Response

Haemolymph samples were preserved immediately after extraction by adding 200–300 μL chilled anticoagulant Lillie’s formol calcium (1.3 M formalin, 126 mM calcium acetate) and haemocytes were counted in an Improved Neubauer haemocytometer at 40× magnification (Olympus CX41 RF) within 48 h. To assess bacteraemia levels, 100 μL of haemolymph was sterilely plated onto each of Zobell’s marine and thiosulphate-citrate-bile salts agars (ZMA and TCBS, Thermofisher), which were incubated at 26 °C for 48 h prior to colonies being counted. Shell necrosis was visually noted as present/absent during dissection.

### 4.8. Nutritional Health Response

Nutritional responses were assessed via Brix index (Hanna Refractometer H196801) and hepatopancreas index (the ratio of hepatopancreas wet weight to lobster wet weight [55,56]).

### 4.9. Haemolymph Biochemical Response

Haemolymph pH was measured using a Radiometer Analytical pH meter PHM210 with micro-electrode B10C162, following which the haemolymph was spun at 10,000× *g* for 5 min (Sigma Microcentrifuge 1–14). The supernatant was snap frozen in liquid nitrogen, stored at −80 °C and sent to Crustipath Laboratories, Canada, for analysis using a Cobas c501 automated biochemistry analyser (Roche Diagnostics Corporation, Indianopolis, IN, USA) as described by Day et al. [21] and Fitzgibbon et al. [22]. Sodium (Na^+^), chloride (Cl^−^), and potassium (K^+^) were measured using an Ion-Selective Electrode, whilst Mg and bicarbonate (bicarb) were measured photometrically. The minerals calcium (Ca) and phosphorous (P); metabolites glucose (Gluc), lactate (Lact), cholesterol (Chol), triglycerides (Trig), total protein (TP), albumin (Alb), globulin (Glob), urea, and uric acid (UA); and enzymes lipase (Lip), amylase (Amy), alanine aminotransferase (ALT), aspartate aminotransferase (AST), alkaline phosphatase (ALP), sorbital (SDH), glutamate dehydrogenases (GD) and gamma-glutamyl transferase (GGT) were measured photometrically. Osmolality was measured on a Micro-Osmette (Precision Systems Inc., Natick, MA, USA) via freezing point depression.

### 4.10. PST Analysis

Paralytic shellfish toxins in the hepatopancreas were analysed at the Cawthron Institute, New Zealand by LC-MS/MS (Waters Acquity UPLC i-Class system coupled to a Waters Xevo TQ-S triple quadrupole mass spectrometer with electrospray ionization), following the methods of Boundy et al. [51] and Turner et al. [52], with minor variations as detailed in Turnbull et al. [8]. Results were calculated using Food and Agriculture Organisation of the United Nations (FAO) toxicity equivalency factors [57]. Results reported as part of this study were corrected based on spike recoveries observed for the different sample matrices analysed. The limit of reporting for each PST analogue differed for each matrix tested.

### 4.11. Gill Histology

Histopathological analysis of gill tissues was conducted at the Animal Health Laboratory, Department of Primary Industries, Parks, Water and Environment in Tasmania. Gills stored in ethanol were embedded in paraffin, cut at 5 µm thickness, mounted and stained with haematoxylin and eosin using standard techniques. All slides were read by the same American College of Veterinary Pathologists (ACVP) board-certified veterinary pathologist.

### 4.12. Data Analysis

Statistical analyses were performed using R Software (R Core Development Team version 3.6, April 2019). Continuous datasets were checked for normality and homoscedasticity using the Shapiro–Wilk test and Levene’s test respectively, with appropriate transformations if necessary (no transformation: SFC, haemocyte count, brix, pH, Na, Cl, TP, Glob, Alb:Glob (A:G), and GD; log transformations: time to right, Ca, Gluc, Chol, and Alb; square root transformations: P, bicarb, Trig, and UA). Analysis of variance was used to test for significant differences between groups for data with normal distributions, followed by post-hoc analysis using Tukey honestly significant difference (HSD) tests. Prior to transformations, P, Lact, bicarb, Trig, UA concentrations that were reported as less than the level of detection (LOD) were replaced with 0.5* LOD (n = 1, 12, 1, 2, 1 respectively). Two-way random permutation tests were used to test for significant differences in continuous data that could not be transformed to a normal distribution (bacterial counts on ZMA and TCBS, K, Na:K, Mg, Lact and measured osmolality).

Ordinal logistic regression was used to test for significant differences between discrete and ordinal datasets (reflex impairment score and vitality respectively), with *p* values calculated by comparing the t-value against the standard normal distribution. Ordinal chi-squared analysis was used to test for association between vitality and RIS. Significant differences between groups in binary datasets (necrosis and gill parasites) were tested using logistic regression.

Analytes where most of the data were below the LOD were not tested for significant differences between groups (creatinine, urea, ALT, ALP, AMY, AST, GGT, and SDH). Differences were considered statistically significant when *p* values < 0.05.

## Figures and Tables

**Table 1:** Summary of differences in behavioural, reflex, immunological, and blood chemistry parameters between control and exposed *J. edwardsii* (Treatment); across days; and for the interaction between treatment and days, as measured by ANOVA, ordinal logistic regression (OLR) or logistic regression (LR). Significant differences are marked with asterisks (* *p* = 0.05–0.01, ** *p* = 0.01–0.001, *** *p* < 0.001). ZMA: Zobell’s marine agar, TCBS: thiosulphate-citrate-bile salts agar.

Variable	Treatment	Days	Treatment: Days	Two-Way ANOVA, OLR, or LR
Behaviour				
Vitality				NS
Time to right				NS
Reflex Impairment Score (RIS)				NS, vitality and RIS sig. related (*p* < 0.001)
Specific feed intake		***		F = 12.3, week 1 higher than weeks 2 and 3
Immune Response				
Haemocyte count		***		F = 18.0, weeks 1 and 2 lower than week 3
Bacteraemia on ZMA media				NS
Bacteraemia on TCBS media				NS
Necrosis				NS
Nutritional				
Hepatopancreas Index				NS
Brix				NS
Hemolymph/Biochemical				
pH	*			F = 4.0, control higher than low exp treatment
Sodium				NS
Potassium				NS
Sodium:potassium				NS
Chloride				NS
Magnesium				NS
Bicarbonate				NS
Calcium				NS
Phosphorus				NS
Glucose				NS
Lactate				NS
Cholesterol		***		F = 8.8, week 1 lower than week 3
Triglyceride		*	*	F = 7.2 (days), week 1 lower than week 3F = 3.5 (treatment:days)
Total protein		*		F = 4.9, week 1 lower than week 3
Albumin				NS
Globulin				NS
Albumin:globulin				NS
Uric acid				NS
Lipase	**			F = 7.4, control is lower than both exposed treatments
Glutamate dehydrogenase				NS
Measured osmolality				NS

## Data Availability

The data presented in this study are available in the supplementary material.

## Supplementary Materials

The following are available online at https://www.mdpi.com/2072-6651/13/2/129/s1, Table S1: The median and standard deviation of behavioural, immunological and biochemical parameters measured in control and PST exposed *J. edwardsii* over different time periods, Table S2: Frequency table of reflex impairment scores (RIS) for *J. edwardsii* control low and high exposure treatment groups (0, 1 × 10^5^ and 2 × 10^5^ cells *A. catenella* respectively) on days 0, 7 and 21, Table S3: Reflex impairment scores in relation to vitality in *J. edwardsii.* All treatment groups combined.
