# Supplementary material for: Lobster Supply Chains Are Not at Risk from Paralytic Shellfish Toxin Accumulation during Wet Storage
Source: Toxins (Basel). 2021 Feb 9;13(2):129. doi: 10.3390/toxins13020129 (PMC7916109; doi:10.3390/toxins13020129)
Supplement: Supplementary file 1 [file toxins-13-00129-s001.pdf]

Supplementary Materials: Lobster supply chains are not at risk from paralytic shellfish toxin accumulation during wet storage

Alison Turnbull, Andreas Seger, Jessica Jolley, Gustaaf Hallegraeff, Graeme Knowles and Quinn Fitzgibbon

Table S1. The median and standard deviation of behavioural, immunological and biochemical parameters measured in control and PST exposed *J. edwardsii* over different time periods

**Day**	**0**		**7**			**21**	
**Treatment**	**Control**	**Control**	**Low Exposed**	**High Exposed**	**Control**	**Low Exposed**	**High Exposed**
PST (mg STX.2HCL equiv. kg^{-1})	0.03 ± 0.01	0.03 ± 0.02	0.04 ± 0.03	0.02 ± 0.01	0.01 ± 0.01	0.01 ± 0.01	0.02 ± 0.0
Time to right	6.7 ± 6.9	3.4 ± 3.4	5.5 ± 9.9	5.9 ± 1.3	2.6 ± 1.5	3.6 ± 1.8	1.6 ± 0.7
Haemocyte count (′ 000 cells mL^{-1})	120 ± 80	120 ± 40	210 ± 80	220 ± 50	310 ± 130	250 ± 110	290 ± 80
Bacteraemia ZMA (cells mL^{-1})	755 ± 1873	906 ± 1828	275 ± 630	268 ± 361	37 ± 72	335 ± 110	165 ± 80
Bacteraemia TCBS (cells mL^{-1})	246 ± 628	319 ± 438	104 ± 173	234 ± 358	46 ± 108	61 ± 128	339 ± 819
Brix	9.6 ± 4.4	12 ± 3.3	12.5 ± 2.7	13.0 ± 3.3	13.8 ± 3.1	13.0 ± 2.7	13.4 ± 3.3
pH	7.62 ± 0.09	7.62 ± 0.09	7.55 ± 0.04	7.60 ± 0.11	7.59 ± 0.16	7.48 ± 0.08	7.50 ± 0.05
Na^{+} (mmol L^{-1})	504 ± 16	498 ± 10	485 ± 23	492 ± 12	500 ± 12	490 ± 22	500 ± 18

K^+ (mmol L^{-1})	9.0 ± 0.9	8.1 ± 1.3	8.6 ± 0.4	9.0 ± 0.8	8.3 ± 1.2	8.7 ± 0.8	8.2 ± 1.1
Na^+:K^+	56 ± 5	63 ± 11	56 ± 2	55 ± 4	62 ± 12	56 ± 5	61 ± 9
Cl^- (mmol L^{-1})	507 ± 22	499 ± 12	482 ± 32	492 ± 14	506 ± 35	485 ± 31	499 ± 25
Ca^{2+} (mmol L^{-1})	14.4 ± 2.6	15.0 ± 2.6	14.9 ± 2.1	14.7 ± 1.0	15.9 ± 1.4	15.8 ± 1.4	15.6 ± 1.8
P^{3-} (mmol L^{-1})	0.6 ± 0.6	0.7 ± 0.7	1.1 ± 0.9	0.8 ± 0.5	0.9 ± 0.3	1.2 ± 1.0	1.1 ± 0.6
Mg^{2+} (mmol L^{-1})	9.7 ± 1.7	8.8 ± 1.3	9.2 ± 1.3	9.3 ± 0.7	10.7 ± 2.1	10.1 ± 1.4	10.2 ± 2.2
Gluc (mmol L^{-1})	2.0 ± 1.9	2.2 ± 1.0	2.0 ± 1.1	1.7 ± 0.5	1.1 ± 0.5	1.5 ± 0.9	2.7 ± 1.5
Lact (mmol L^{-1})	0.27 ± 0.16	0.26 ± 0.17	0.43 ± 0.20	0.60 ± 0.36	0.37 ± 0.18	0.58 ± 0.41	0.38 ± 0.34
HCO_3^- (mmol L^{-1})	5.4 ± 1.5	3.9 ± 1.8	4.5 ± 0.8	4.4 ± 0.5	5.5 ± 1.7	5.2 ± 0.8	5.4 ± 1.2
Chol (mmol L^{-1})	0.41 ± 0.31	0.57 ± 0.22	0.65 ± 0.47	0.47 ± 0.13	0.81 ± 0.27	0.56 ± 0.15	0.70 ± 0.39
Trigly (mmol L^{-1})	0.37 ± 0.33	0.61 ± 0.20	0.70 ± 0.38	0.55 ± 0.18	0.87 ± 0.31	0.56 ± 0.14	0.70 ± 0.38
TProt (g L^{-1})	50 ± 32	60 ± 23	64 ± 20	61 ± 18	78 ± 21	67 ± 20	70 ± 22
Alb (g L^{-1})	7.3 ± 4.3	6.4 ± 1.5	7.3 ± 3.8	7.8 ± 2.2	11.3 ± 4.2	8.1 ± 2.3	7.8 ± 2.6
Glob (g L^{-1})	42 ± 28	53 ± 24	57 ± 21	53 ± 16	67 ± 19	59 ± 20	63 ± 21
A:G	0.20 ± 0.07	0.14 ± 0.06	0.14 ± 0.07	0.15 ± 0.04	0.18 ± 0.06	0.15 ± 0.06	0.13 ± 0.05
UA (μmol L^{-1})	16 ± 20	14 ± 17	26 ± 23	24 ± 18	30 ± 15	23 ± 13	32 ± 15
Lip (g L^{-1})	3.0 ± 0.8	4.1 ± 1.3	5.4 ± 1.9	4.8 ± 1.3	4.3 ± 0.8	5.0 ± 1.3	5.0 ± 1.3
GD (g L^{-1})	9.6 ± 3.5	19.6 ± 10.4	15.0 ± 8.6	16.5 ± 4.2	16 ± 6.0	16.8 ± 5.4	20.1 ± 8.4

Measured Osmolality	1043 ± 41	1029 ± 34	1010 ± 28	1033 ± 25	1007 ± 49	1034 ± 15	1020 ± 31

Table S2. Frequency table of reflex impairment scores (RIS) for *J. edwardsii* control low and high exposure treatment groups (0, 1x10^5 and 2x10^5 cells *A. catenella* respectively) on days 0, 7 and 21.

			**Days**	
**RIS**	**Treatment**	**0**	**7**	**21**
1	Control	1	1	0
	Low exposure		3	1
	High exposure		3	3
2	Control	1	3	2
	Low exposure		2	1
	High exposure		0	1
3	Control	2	1	2
	Low exposure		2	2
	High exposure		1	0
4	Control	2	1	0
	Low exposure		0	0
	High exposure		1	0
5	Control	1	1	2
	Low exposure		0	3
	High exposure		0	1
6	Control	0	0	1
	Low exposure		0	0
	High exposure		1	1

Table S3. Reflex impairment scores in relation to vitality in *J. edwardsii.* All treatment groups combined.

	**Reflex Impairment Score**					
**Vitality**	**1**	**2**	**3**	**4**	**5**	**6**
3	0	0	0	0	1	2
4	1	0	1	2	3	0
5	11	9	9	2	4	1
